# In the September 2010 issue

**DOI:** 10.1590/S1980-57642010DN40300001

**Published:** 2010

**Authors:** Ricardo Nitrini

**Affiliations:** Editor-in-Chief

In this issue we have published four reviews and 11 original papers.

**Brucki** investigated the association between illiteracy and dementia, a complex issue where a host of different factors such as low cognitive reserve, poor control of cerebrovascular disease risk factors, difficulties in cognitive evaluation, and poor adaptation of neuropsychological tests interact, increasing the prevalence of dementia in illiterates in epidemiological studies.

**Schafirovits-Morillo and Suemoto** analyzed the challenge of providing the best care to patients and their caregivers in advanced-stage dementia. This issue, largely overlooked until recently, is attracting growing interest from clinicians and researchers. The authors stressed the importance of acknowledging cultural differences, and of the approaches to determine patient’s opinions on end of life and palliative care issues from the early phase of dementia.

**Nagaoka and Ortiz** performed a critical review of the literature on apraxia, from its original concept and description to current classification, pathophysiology and clinical-anatomical correlations.

Two articles, one review and one original paper, present data on the anatomy of the brain, and on behavioral and cognitive functions of a New World monkey, *Cebus sp*.

**Aversi-Ferreira** and colleagues from the Federal University of Goiás, Brazil, together with Nishijo from the Laboratory of Neurosciences and Behavioral of Primates of the University of Toyama, Japan, review the cognitive and behavioral symptoms and signs related to the human parietal lobes. The authors also review the anatomy of the human parietal cortex comparing it with the parietal lobes of other primates. A relevant contribution is the conclusion that the New World capuchin monkey (*Cebus libidinosus*) may be a more viable alternative for studies of primate brain functions, mainly because of its medium weight (1.1 to 3.3 Kg for the adult monkey), which makes it less expensive to maintain in the laboratory than Old World primates.

**Borges et al.**, from Goiás State University, Brazil, studied the brain of the *Cebus apella* and disclosed that the prefrontal areas represent almost 20% of the cerebral volume of this primate, a very similar proportion to that found in human brain. The authors conjecture whether the greater size of the frontal lobe of *C. apella* is related to the elaborate cognitive functions of this animal, particularly its creativity in the usage of fruit as bait and the ability to interpret images, including a theory of mind.

**Stella et al.** investigated the correlation between the severity of dementia and the occurrence and severity of apathy, using the Apathy Evaluation Scale and the Neuropsychiatric Inventory for rating apathy and the Mini-Mental State Examination, Clinical Dementia Rating, and Global Deterioration Scale for dementia severity.

**Ribeiro et al.** compared the performance of healthy elderly of three schooling levels (low, intermediate and high) in the generation of visual inferences from pictures of different levels of complexity. They concluded that visual complexity interferes with the ability to make inferences in low- and medium-educated but not in high-educated individuals.

**Cecato et al.** evaluated the repetition of phrases in elderly control subjects and in Alzheimer’s disease patients using phrases from two tests: the Mini-Mental State Examination (MMSE) and the MoCA (Montreal Cognitive Assessment). Both control subjects and patients were able to repeat the MMSE phrase, while up to 60% of patients with Alzheimer’s disease were unable to correctly repeat one of the two phrases of the MoCA. They concluded that the repetition phrase task in the MMSE is less sensitive for detecting mild language decline in AD patients.

**Banhato et al.** employed the Short Form of the Wechsler-III Scale in 168 elderly individuals to evaluate the sensitivity and specificity for detecting cognitive impairment. They found high accuracy with the use of cut-off scores adjusted for age.

**Pereira et al.** followed a cohort of healthy elderly for three years using two cognitive tests and a functional questionnaire. There was discrete, albeit significant, decline on both cognitive and functional evaluation tests in this relatively short follow-up period, highlighting the relevance of longitudinal cognitive screening studies in understanding age-associated cognitive decline.

**Netto et al.** performed a systematic review of the literature to assess the effectiveness of interventions designed to improve working memory in adults. They found only three randomized clinical trials, and reported that interventions were more effective in patients with neurological diseases than in healthy adults. The main conclusion was that there is a need for further studies to verify the effectiveness of WM intervention programs in aging and in dementia.

**Diel et al.** investigated sociodemographic characteristics of caregivers and non-caregivers attending a support unit for family members of patients with dementia. Most of the participants were women and caregiver burden was rated as being up to moderate. Those with high educational level had higher burden as evaluated by the Zarit Burden Interview.

**Balieiro et al.** analyzed the relationship between caregiver distress and behavioral and psychological symptoms in mild Alzheimer’s disease, using the Neuropsychiatric Inventory (NPI) and the Caregiver Distress Index (CDI). They found strong correlation between NPI and CDI total scores, but when particular neuropsychiatric symptoms or their characteristics such as frequency and severity, were analyzed, the associations were not well defined, suggesting that further studies in larger samples are necessary.

**Nazir and Mushtaq** reported the safety and tolerability of the rivastigmine patch in a clinical setting. Thirty patients were included in a prospective observational study. Adverse events occurred in 20%, causing discontinuation of the treatment in 3 patients (10%), while cognitive or functional improvement was observed in two-thirds of the patients.

**Ribas et al.** investigated attention capacity in air traffic controllers who were in the profession for more than 10 years, comparing their performance with those working less than 10 years and with controls. They found that air traffic controllers in the job for more than 10 years had better performance on attention and concentration tests than did normal controls.

**Ricardo Nitrini** Editor-in-Chief

